# Host Plant Species Influences the Composition of Milkweed and Monarch Microbiomes

**DOI:** 10.3389/fmicb.2022.840078

**Published:** 2022-02-24

**Authors:** Thorsten E. Hansen, Laramy S. Enders

**Affiliations:** Entomology Department, Purdue University, West Lafayette, IN, United States

**Keywords:** milkweed, monarch, microbiome, rhizosphere, phyllosphere

## Abstract

Plants produce defensive chemicals for protection against insect herbivores that may also alter plant and insect associated microbial communities. However, it is unclear how expression of plant defenses impacts the assembly of insect and plant microbiomes, for example by enhancing communities for microbes that can metabolize defensive chemicals. Monarch butterflies (*Danaus plexippus*) feed on milkweed species (*Asclepias* spp.) that vary in production of toxic cardiac glycosides, which could alter associated microbiomes. We therefore sought to understand how different milkweed species, with varying defensive chemical profiles, influence the diversity and composition of monarch and milkweed (root and leaf) bacterial communities. Using a metabarcoding approach, we compared rhizosphere, phyllosphere and monarch microbiomes across two milkweed species (*Asclepias curassavica*, *Asclepias syriaca)* and investigated top-down effects of monarch feeding on milkweed microbiomes. Overall, monarch feeding had little effect on host plant microbial communities, but each milkweed species harbored distinct rhizosphere and phyllosphere microbiomes, as did the monarchs feeding on them. There was no difference in diversity between plants species for any of the microbial communities. Taxonomic composition significantly varied between plant species for rhizospheres, phyllospheres, and monarch microbiomes and no dispersion were detected between samples. Interestingly, phyllosphere and monarch microbiomes shared a high proportion of bacterial taxa with the rhizosphere (88.78 and 95.63%, respectively), while phyllosphere and monarch microbiomes had fewer taxa in common. Overall, our results suggest milkweed species select for unique sets of microbial taxa, but to what extent differences in expression of defensive chemicals directly influences microbiome assembly remains to be tested. Host plant species also appears to drive differences in monarch caterpillar microbiomes. Further work is needed to understand how monarchs acquire microbes, for example through horizontal transfer during feeding on leaves or encountering soil when moving on or between host plants.

## Introduction

In the context of plant-insect interactions, microbial partnerships can become powerful adaptive weapons in the ongoing arms race between herbivores and their host plants. Many microbes provide essential nutrients that are lacking in the host’s diet, allowing insect herbivores to expand their host plant range and take advantage of novel ecological niches (Hammer and Bowers, 2015). By aiding in the breakdown of plant defensive chemicals, detoxifying microbial symbionts can also facilitate the insect host’s ability to feed on plants that would normally be deadly when consumed (Itoh et al., 2018). Plants also utilize microbial partnerships to resist or tolerate insect damage. For example, rhizobacteria facilitate nutrient mobilization, acquisition and translocation from plant roots to shoots (Dotaniya and Meena, 2015; Pii et al., 2015), which can boost plant growth and improve recovery from insect feeding damage (Hubbard et al., 2019; Goswami and Suresh, 2020). Rhizosphere bacteria are also well known for their ability to prime plant defenses against insect attackers and inducing systemic resistance (ISR), which can lead to upregulation of plant hormones and the production of defensive chemicals (Thaler et al., 2012; Mauch-Mani et al., 2017; Pineda et al., 2017).

Microbes not only directly influence plant-insect interactions, but exist in complex interacting insect- and plant-associated communities (i.e., microbiomes) that are shaped by multiple abiotic and biotic factors. Shifts in environmental pH, temperature, moisture and nutrient availability can all affect the diversity and structure of plant and insect microbiomes (Bais et al., 2006; Douglas, 2015; van der Voort et al., 2016; Hammer et al., 2017; Van Agtmaal et al., 2017). However, arguably one of the most common selection pressures acting on plant- and insect-associated microbial communities are plant defenses. When plants are fed on, they release an array of compounds that can broadly affect root and leaf associated microbiomes, including secondary metabolites that deter feeding and prevent herbivore damage. For example, aboveground insect feeding can induce the production of root exudates (e.g., sugars, photosynthates, phytotoxins) belowground that affect rhizosphere microbial communities (van Dam and Heil, 2011). Root exudates can impose a variety of affects: they can be carbon sources, act as attractants/repellants, promote growth, and/or have antibiotic effects on specific microbes (Pang et al., 2021). Plant chemical defenses are unique in that they provide direct protection against herbivores (Mithöfer and Boland, 2012; Raguso et al., 2015) but can also have antimicrobial affects that may impose selection on the microbiomes associated with plant rhizospheres (Baetz and Martinoia, 2014; Musilova et al., 2016) and insect herbivores (Hansen and Moran, 2014; Douglas, 2015). However, not all microbes will be negatively affected by secondary plant compounds. Many have evolved systems to degrade antimicrobials or use them as a carbon source (Musilova et al., 2016; Priyadharsini et al., 2016; Tyc et al., 2017). As a result, it is unclear to what extent secondary plant compounds select for microbial communities with enriched chemical detoxification and whether there are cascading effects on plant-insect interactions.

Investigation of relationships between highly specialized herbivores and the chemically defended hosts plants they feed on is one approach to better understand how plant defenses shape plant- and insect-associated microbial communities. Milkweeds (*Asclepias* spp.) and their complex of specialist herbivores are an ideal system to explore factors driving microbiome variation across host plant species. Milkweeds contain cardenolides, highly toxic steroidal secondary compounds, which vary across milkweed species in concentration, diversity, and composition (Agrawal et al., 2012). Cardenolides not only affect the fitness of specialist herbivores that feed on milkweeds, but also likely impact the microbial communities of both the plant host and insect attackers. Cardenolides can exhibit antimicrobial activity (Jacobsohn and Jacobsohn, 1985; Akhtar et al., 1992; Bertol et al., 2011) and are produced in response to microbial infection (Agrawal et al., 2012). Cardenolides are also systemically expressed throughout all milkweed tissues from roots, shoots, and seeds (Agrawal et al., 2012) and therefore have the potential to widely influence plant-associated microbiomes, in addition to the microbiomes of insect herbivores that consume these plant tissues. However, little is currently known about the microbiomes of milkweeds and their insect herbivores or the extent to which cardenolides potentially shape surrounding microbial communities.

In this study we explore the diversity and composition of milkweed- and monarch-associated microbiomes in milkweed species that vary in defensive chemical profiles. Specifically, we addressed the following questions: (1) Does microbiome diversity and composition associated with host plant tissues and insect herbivores vary across milkweed species? (2) Does herbivore feeding cause changes in root- and leaf-associated microbiomes? and (3) To what extent do herbivore and plant- associated microbial communities have common or shared microbial taxa? We used a 16s rRNA metabarcoding approach to characterize bacterial microbiomes associated with monarch butterfly caterpillars (*Danaus plexippus*) and both above and belowground plant microbiomes (i.e., rhizosphere, phyllosphere) across two milkweed species, the common milkweed (*Asclepias syriaca*) and the tropical milkweed (*Asclepias curassavica*). The defensive chemical profiles of both milkweed species are distinct, as *A. syriaca* has lower total concentration and polarity of cardenolides compared to *A. curassavica*, in both shoots and roots (Rasmann and Agrawal, 2011). We hypothesized differences in defensive chemical profiles would select for divergent plant and herbivore microbial communities, in particular milkweed species with higher concentrations of cardenolides (*A. curassavica)* would select for overall lower microbial diversity in rhizosphere, phyllosphere, and monarch microbiomes. We also predicted that monarch feeding would induce changes in the microbial communities of the milkweed rhizosphere and phyllosphere and this effect would be greatest in communities associated with *A. curassavica*. And finally, we predicted that monarch and milkweed phyllosphere microbiomes would be more similar than monarch-rhizosphere or phyllosphere-rhizosphere comparisons.

## Materials and Methods

### Plant and Insect Materials

Milkweed seeds from two species, *A. syriaca* and *A. curassavica*, were purchased from Everwilde Farm Inc. Prior to germination and planting, *A. syriaca* seeds were cold stratified at 4°C on moist filter paper for 2–3 weeks, surface sterilized with 5% bleach, germinated at room temperature (21 ± 1°C) inside a growth chamber, and planted in autoclaved potting soil. Seeds of *A. curassavica* were not cold stratified and only surface sterilized. Unlike *A. syriaca* whose seeds overwinter in freezing soil (Borders and Lee-Mäder, 2014), *A. curassavica* does not require cold conditions for germination because they are native to tropical Mesoamerica and South America (Woodson, 1954). Finally, monarch eggs were obtained from Shady Oak Butterfly Farm, Inc. Once larvae hatched, they were fed on *A. syriaca* leaves until they reached 2nd instar and were used in the experiment described below.

Milkweed seedlings were grown in 10 cm diameter pots under controlled greenhouse conditions (14 h daylight, 26°C day: 20°C night) for 35–40 days from July to August 2018. Once the seedlings reached leaf stage 2–3, they were transferred to a growth chamber (14 h daylight, 26°C day: 20°C night) for the remainder of the experiment when the insect infestation treatment was applied. Each pot received 12 ml of Hoagland solution as an initial fertilization treatment. All plants were bottom watered with Milli-Q “ultrapure” water to prevent introduction of minerals that could influence soil microbial composition, minimize disturbance, and avoid leaching of microbes that can occur with top watering. Milkweed root and leaf microbiomes examined in this study are therefore derived from a combination of internal seed microbiota, natural colonization of microbes in the open greenhouse over time (9 weeks of growth) and microbes introduced by monarch caterpillar feeding.

### Experimental Design and Sample Collection

To examine the extent to which key factors, including host plant species and response to herbivore feeding, influence milkweed and monarch microbial communities, we conducted an experiment comparing two milkweed species (*A. syriaca* and *A. curassavica*) under two herbivore treatments (infested or un-infested). A total of 28 plants (*n* = 14/host plant species) were grown to leaf stage 3–4 (∼ 35–40 days after planting), half the replicates were infested with a 2nd instar monarch larva (*n* = 7/host plant species) and the other half were not infested (*n* = 7/host plant species). The whole plant was enclosed with a custom-made mesh cage (27 cm long × 10.5 cm wide) to prevent insect escape and placed in a growth chamber. Monarch larvae were allowed to feed for 4 days, after which samples were collected for further analysis of whole insect, rhizosphere and phyllosphere microbial communities. The infestation period (4 days) was chosen based on (1) previous research showing monarch herbivory causes significant induction of plant defenses (e.g., cardenolide production) that peaks 2–3 days after feeding and can remain elevated for 10 days (Agrawal et al., 2012) and (2) preliminary feeding assays used to determine an optimal feeding duration that would inflict damage to the plant to induce defenses but avoid complete defoliation of the plant (i.e., leave tissue for analysis). Monarch weights were taken before they were introduced to plants and again after they were removed when the experiment finished.

Prior to DNA extraction, all monarch larvae were surface sterilized with 10% bleach, 70% ethanol, and three washes of autoclaved water. To collect rhizosphere soil microbiomes samples, roots were agitated for 15 min in phosphate-buffered saline (PBS) using a standing shaker to separate soil particles from plant roots. The plant roots were then removed, and the remaining soil and PBS solution was centrifuged at 3,000 rcf for 15 min as described in Hubbard et al. (2019). Leaf tissue used for phyllosphere microbiome analysis was collected by clipping the leaves growing from the second node down from the apical meristem. Leaves were surface sterilized in the same manner as the monarch larvae and stored in sterilized tubes at -80C.

### Microbial Community Characterization and Analysis

Whole monarch larvae were homogenized, and total DNA extracted using the DNeasy extraction kits (Qiagen, Valencia, CA, United States) following the manufacturer’s protocol. Whole caterpillar bodies were used for DNA extraction to include all internal microbes found in the hemolymph or associated with tissues. Rhizosphere bacterial DNA was extracted by first centrifuging samples, removing the PBS supernatant, and transferring the remaining 250 mg soil pellet to bead tubes, at which point DNA was extracted using the Mobio Power Soil DNA Isolation Kit (Mobio Laboratories, Carlsbad, CA, United States) following the manufacturer’s instructions. Phyllosphere bacterial DNA was extracted by first grinding the leaf tissue in liquid nitrogen using a sterilized mortar and pestle, and then transferring 250 mg ground leaf tissue to bead tubes for extraction using the Mobio Power Soil DNA Isolation Kit. Tissues were lysed in beadtubes using a Precellys^®^ 24 homogenizer for a total of 10 min at 6,800 rpm; which was applied as repeated 30 s bursts with 1 min rest periods. All DNA samples were sent to the University of Minnesota Genomics Center (Nils Hasslemo Hall, MN) for Illumina MiSeq sequencing following in-house optimized methods (Gohl et al., 2016). A 250 bp segment, of the V4 region, of the 16S rRNA subunit gene was amplified for insect herbivores, leaves, and soil samples using standard V4 region primers 515F-GTGCCAGCMGCCGCGGTAA and 806R-GGACTACHVGGGTWTCTAAT. All sequence data is available on NCBI SRA database under project number PRJNA786874.

Sample demultiplexing was done by the University of Minnesota Genomics Center with Illumina software, and Trimmomatic (Bolger et al., 2014) and Cutadapt (v 1.13) (Martin, 2011) were used to remove adapters, primer sequences, and low-quality reads. Subsequent sequence processing was performed in Qiime2 (2-2019.10) (Bolyen et al., 2019). Raw reads were processed with the DADA2 pipeline (v 1.10), which filtered and trimmed based on read quality, inferred error rates, merged paired-end reads, removed chimeras, and assigned taxonomy to identified amplicon sequence variants (ASVs) using the Silva reference database (v 132) (Quast et al., 2012). Through this process eukaryotic, mitochondria, chloroplast, Archaea sequences and possible contamination (e.g., aphid derived *Buchnera aphidicola*) were removed. Phylogenetic trees were produced for identified ASVs, which were used to calculate unweighted and weighted unifrac values. After preprocessing, the dataset contained ∼1.2 million reads in 69 samples, averaging 17,552 reads per sample (see Supplementary Table 1 for sequencing summary).

Following sequence processing, all downstream analyses were run in R (v 4.0.1) (R Core Team, 2013). All code for statistical analyses and generation of figures can be found in the Purdue University Github.^1^ Change in monarch weights from the start and end of the experiment were compared using a Welch two sample *t*-test. We compared standard alpha and beta diversity metrics using the phyloseq (v 1.32.0) (McMurdie and Holmes, 2013), vegan (v 2.5.7) (Oksanen et al., 2015), and rstatix (0.6.0) (Kassambara, 2020) packages in R. To compare species richness and evenness (e.g., observed species richness, species evenness, Shannon diversity, Simpson diversity) we used a Kruskal-Wallis rank sum test. Separate tests were run to compare plant species (*A. syriaca* vs. *A. curassavica*) and insect presence (± monarch) for each alpha diversity metric (e.g., observed species richness ∼ plant species; observed species richness ∼ insect presence). Differences in the structure of bacterial communities was assessed through PERMANOVA of beta diversity (Bray-Curtis) and visualized using Principal Coordinates Analysis (PCoA). The package metagenomeSeq (v 1.30.0) (Paulson et al., 2013) was used to normalize read counts through Cumulative Sum Scaling (CSS) prior to analysis of beta diversity. CSS corrects for differences in sampling depth (library size), which can be an issue when comparing microbiomes from different environments/tissues. This normalization technique was applied in previous research comparing microbiomes between soil, leaves, and insects (Hannula et al., 2019). We analyzed plant microbiomes and insect microbiomes separately in order to address two of our central questions: (1) How does microbial community diversity and composition vary across milkweed species? and (2) Does herbivore feeding cause changes to the root and leaf associated microbiomes of milkweed plants? The PERMANOVA model for rhizosphere and phyllosphere microbiomes included the following: plant species, insect presence, and plant species x insect presence. The PERMANOVA model for monarch caterpillar microbiomes included a single factor: plant species. Dispersion across samples was also tested using PERMDISP. We also used ANCOM (v 2.1) (Mandal et al., 2015) to identify microbial families that were differentially abundant in the rhizosphere, phyllosphere and monarch samples when comparing across milkweed species (model: plant species). Finally, we were interested in the extent to which microbes were shared across milkweed roots, leaves and monarchs. We identified shared vs. unique ASVs in rhizosphere, phyllosphere and monarch microbiomes and visualized results using the package *VennDiagram* (v 1.6.20) (Chen, 2018).

## Results

### Monarch, Phyllosphere, and Rhizosphere Microbiomes Differ Across Host Plant Species

**FIGURE 1:**
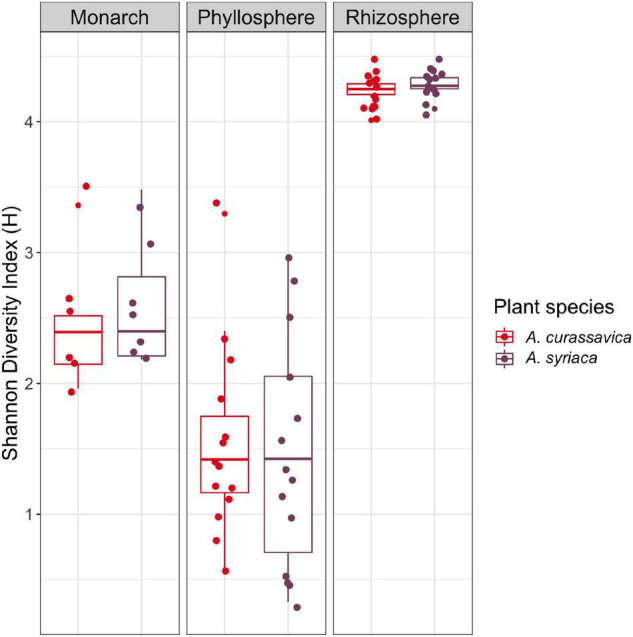
Comparison of Shannon diversity of microbial communities associated with two milkweed species (roots/rhizosphere soil, leaves/phyllosphere) and monarch larva after feeding on the different milkweed species.

We compared bacterial microbiomes to determine if both plant and herbivore associated communities differed depending on milkweed species. In total, there were 2,135 ASVs in the rhizosphere, 412 in the phyllosphere, and 205 for the monarchs. Overall, bacterial alpha diversity was highest in the rhizosphere, followed by the monarch larvae, and finally the phyllosphere (Figure 1 and Supplementary Figure 1). We expected lower diversity for all microbiomes associated with *A. curassavica*, as it is known to have higher concentrations of cardenolides, compared to *A. syriaca.* However, Shannon diversity did not differ between milkweed species for monarch (*H* = 0.3265, *P* = 0.568), phyllosphere (*H* = 0.0760, *P* = 0.783), or rhizosphere communities (*H* = 2.4405, *P* = 0.118) (Figure 1). The results found for Shannon diversity were generally similar to all other alpha diversity measures analyzed (Observed Species Richness, Evenness, Simpson Diversity Index; for details see Supplementary Figure 1 and Supplementary Table 2).

**FIGURE 2:**
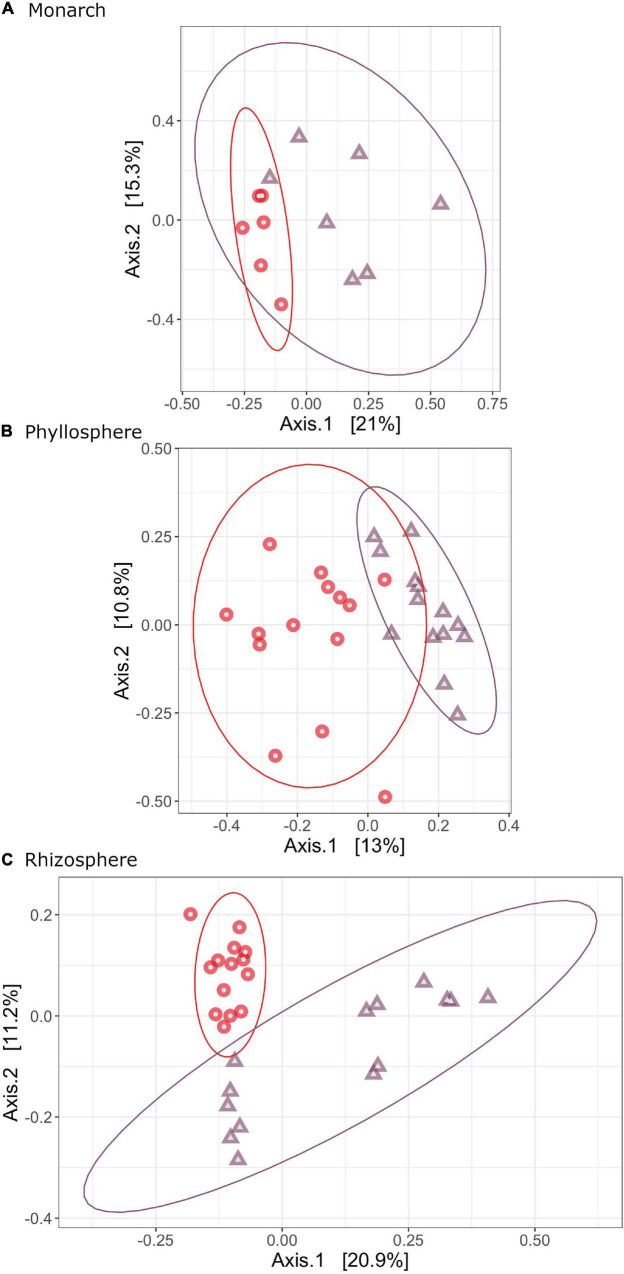
PCOAs of Bray-Curtis dissimilarity showing how bacterial community structure varies across milkweed species for monarch **(A)**, phyllosphere **(B)** and rhizosphere **(C)** bacterial microbiomes. Samples are colored by milkweed species (red, *A. curassavica*; purple, *A. syriaca*).

Significant variation in bacterial community composition was observed for monarchs (*F* = 2.2529, *R*^2^ = 0.1699, *P* = 0.002), phyllosphere (*F* = 2.9971, *R*^2^ = 0.1034, *P* = 0.001), and rhizosphere (*F* = 4.1747, *R*^2^ = 0.1393, *P* = 0.001) (Supplementary Table 3). Taxonomic composition varied significantly between *A. syraica* and *A. curassavica*, with host plant species explaining 16.99% (monarch), 10.34% (phyllosphere), and 13.93% (rhizosphere) of variation between samples (Figure 2). We did not detect differences in dispersion between samples for microbial communities in the monarch, phyllosphere, or rhizosphere (monarch: *F* = 0.4109, *P* = 0.505; phyllosphere: *F* = 0.2435, *P* = 0.609; rhizosphere: *F* = 0.4151, *P* = 0.592) (Supplementary Table 4).

**FIGURE 3:**
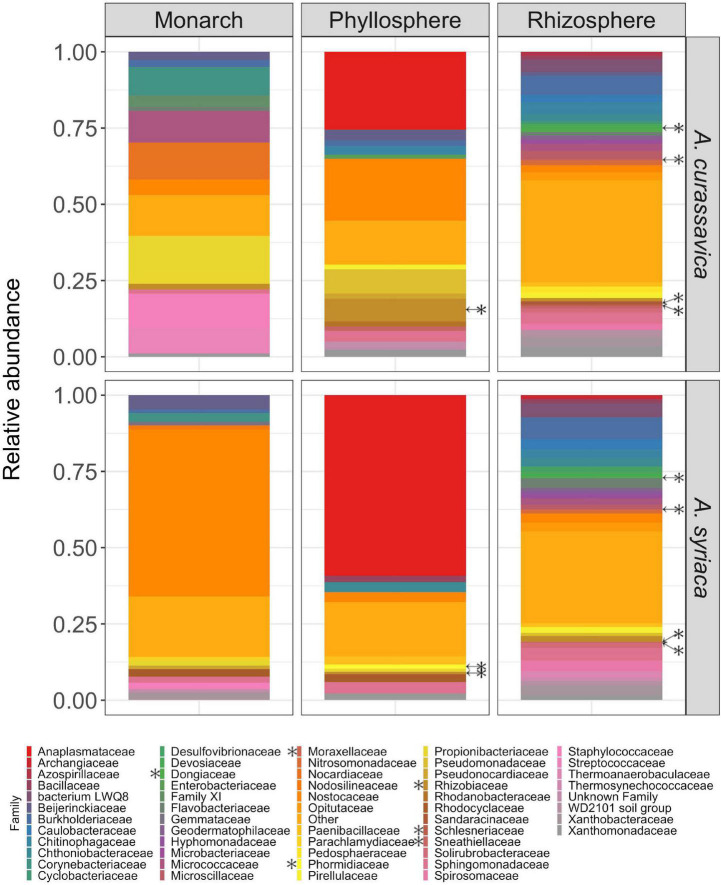
Differences in the taxonomic composition of rhizosphere, phyllosphere and monarch associated microbial communities across milkweed host plant species. *Indicates significant enrichment of bacterial family in microbiomes, across plant species treatments, based on ANCOM analysis (Supplementary Table 5 *p* < 0.05).

To further investigate differences between microbiomes associated with different milkweed plant species, we identified microbial families of interest and differentially abundant microbial families. Monarch microbiomes generally consisted of a mixture of families in low relative abundance (<1%) with a single dominant family that varied by host plant; for monarchs feeding on *A. curassavica* the most abundant family was Micrococcaceae (10.38%), and on *A. syriaca* Nostocaceae (40.88%) the most abundant family (Figure 3 and Table 1). In addition, we detected two monarch associated bacterial families with higher relative abundance when caterpillars fed on *A. syriaca*: Beijerinkiaceae and Nostocaceae (Table 1). Phyllosphere microbiomes were generally dominated by the family Anaplasmataceae (46.84% in *A. curassavica* and 74.02% in *A. syriaca)* and all ASVs within this family were identified as belonging to the genus *Wolbachia*. Where milkweed phyllospheres differed was in the overall composition of additional lower abundance bacterial families. *A. curassavica* phyllosphere microbiomes contained Nostocaceae (10.62%), Rhizobiaceae (7.95%), Sphingomonadaceae (6.31%), and Pseudomonadaceae (4.77%), while in contrast *A. syriaca* leave microbiomes harbored additional bacterial families present at lower levels (Figure 3 and Table 1). Statistically significant differences between phyllospheres include Phormidiaceae (*W* = 47, *P* < 0.05), which were completely absent from *A. curassavica* leaves, and Rhizobiaceae (*W* = 53, *P* < 0.05), which were more abundant in the leaves of *A. curassavica* (Figure 3 and Supplementary Table 5). Finally, across both plant species the three most abundant microbial families in the rhizosphere were Burkholderiaceae, Sphingomonadaceae, and Xanthobacteraceae, all found in relatively similar abundances (Figure 3 and Table 1). However, composition of lower abundance (∼1%) bacterial families in the rhizosphere differed between plant species (Table 1). Statistically significant differentially abundant families in the rhizosphere were Dongiaceae (*W* = 175, *P* < 0.05), Moraxellaceae (*W* = 219, *P* < 0.05), and Schlesneriaceae (*W* = 155, *P* < 0.05), which were more abundant in *A. curassavica* rhizospheres, while Sneathiellaceae (*W* = 150, *P* < 0.05), was more abundant in *A. syriaca* rhizospheres (Figure 3 and Supplementary Table 5).

**TABLE 1:** Subset of most abundant bacterial families associated with microbiomes of two milkweed plant species (roots/rhizosphere soil, leaves/phyllosphere) and monarch caterpillars after feeding on each species.

	Monarch		Phyllosphere		Rhizosphere	
Bacterial family	*A. curassavica*	*A. syriaca*	*A. curassavica*	*A. syriaca*	*A. curassavica*	*A. syriaca*
Anaplasmataceae	0.00	0.63	46.84	74.02	0.00	0.00
Azospirillaceae	0.23	0.00	0.051	0.023	1.46	0.96
Bacillaceae	0.83	0.79	0.24	1.52	1.33	1.36
Bacterium LWQ8	0.00	0.13	0.011	0.00	3.24	2.99
Beijerinkiaceae	2.45	7.91	3.13	0.89	1.48	1.35
Burkholderiaceae	2.98	1.46	1.27	0.085	10.54	10.72
Caulobacteraceae	0.44	0.58	0.39	0.15	3.26	3.96
Chitinophagaceae	0.19	0.16	1.36	0.080	4.01	3.06
Chthoniobacteraceae	0.17	0.72	0.21	1.60	2.77	1.84
Devosiaceae	0.19	0.11	0.16	0.18	1.43	1.92
Dongiaceae	0.00	0.20	0.50	0.39	1.39	0.32
Flavobacteriaceae	1.84	0.88	0.033	0.023	0.69	2.47
Geodermatophilaceae	0.00	1.08	0.37	0.74	1.58	1.25
Hyphomonadaceae	0.21	0.00	0.17	0.00	1.02	0.94
Microbacteriaceae	0.29	0.13	0.88	0.28	1.69	1.65
Micrococcaceae	10.38	1.55	0.70	0.32	2.11	2.08
Microscillaceae	0.87	0.11	0.11	0.063	2.94	1.89
Nitrosomonadaceae	0.12	0.067	0.037	0.080	1.86	1.58
Nostocaceae	3.67	40.88	10.62	2.57	1.50	1.34
Opitutaceae	0.00	0.11	0.29	0.011	2.98	2.98
Paenibacillaceae	0.19	0.54	0.25	2.01	1.60	1.71
Pedosphaeraceae	0.68	0.00	0.16	0.069	1.37	1.05
Pirellulaceae	0.64	0.90	0.76	0.075	1.85	2.38
Pseudomonadaceae	0.91	0.45	4.77	0.29	0.83	1.05
Rhizobiaceae	1.43	0.81	7.95	0.40	1.57	2.98
Solirubrobacteraceae	0.12	0.00	0.077	0.12	1.55	1.97
Sphingomonadaceae	1.26	3.41	6.31	3.64	4.97	6.28
Spirosomaceae	0.87	0.00	0.033	0.11	2.19	3.39
Uncultured	0.00	0.11	0.077	0.10	1.06	1.07
Uncultured Clostridia	0.00	0.00	0.10	0.00	1.00	0.24
WD2101 soil group	0.00	0.00	0.051	0.063	2.36	1.82
Xanthobacteraceae	1.22	2.94	0.14	0.63	4.08	4.88
Xantomonadaceae	0.97	0.43	1.25	1.29	3.79	1.81

*Values are relative abundance of reads, across samples, per treatment.*

### Monarch Feeding Did Not Significantly Alter Rhizosphere or Phyllosphere Microbiomes

**FIGURE 4:**
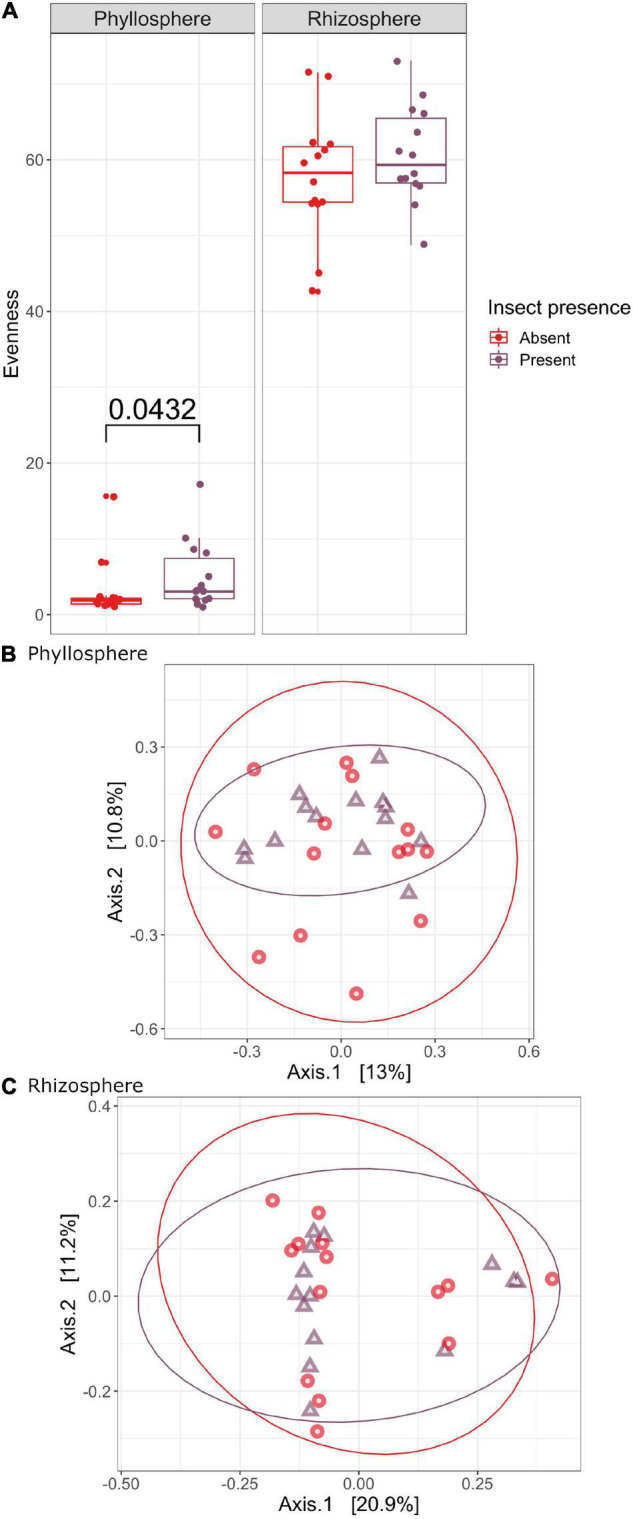
The effect of monarch feeding on evenness **(A)** and community composition of milkweed phyllosphere **(B)** and rhizosphere **(C)** bacterial microbiomes. PCOAs of Bray-Curtis dissimilarity showing how bacterial community structure varies between plants with insects present and absent. Samples are colored by insect presence (red, un-infested control, purple, monarch infested).

To determine the extent to which monarch feeding induced changes in the rhizosphere and phyllosphere microbiomes of milkweed host plants, we compared infested plants to un-infested controls. Overall, bacterial species richness did not vary with monarch feeding (Supplementary Figure 2 and Supplementary Table 2), but there was a trend for greater evenness of microbial taxa in the phyllosphere of un-infested plants (*H* = 4.0872, *P* = 0.0432) (Figure 4). We also found no difference in taxonomic composition of milkweed microbiomes fed on by monarchs compared to those that were not (phyllosphere: *F* = 1.0440, *R*^2^ = 0.0360, *P* = 0.390; rhizosphere: *F* = 0.7841, *R*^2^ = 0.0261, *P* = 0.747) (Figure 4 and Supplementary Table 3). There were no differences in dispersion between samples where insects were present or absent for the microbial communities in the phyllosphere or rhizosphere (phyllosphere: *F* = 0.0803, *P* = 0.775; rhizosphere: *F* = 2.1118, *P* = 0.165) (Supplementary Table 4). Interestingly, the change in monarch weight was significantly different as monarchs feeding on *A. curassavica* had lower weights than those feeding on *A. syriaca* by the end of the experiment (Supplementary Figure 3 and Supplementary Table 6).

### Monarch Microbiomes Have More in Common With the Rhizosphere Than Phyllosphere Microbiome

**FIGURE 5:**
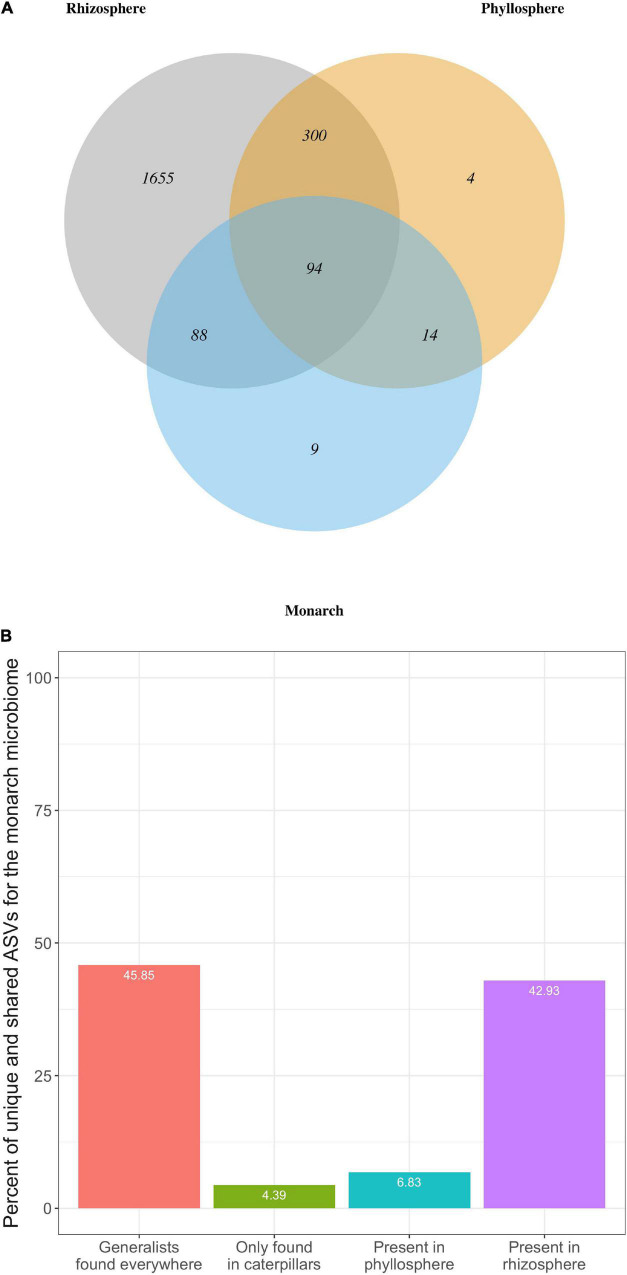
**(A)** Venn Diagram showing total number of microbial taxa (ASVs) that are shared across monarch, phyllosphere, and rhizosphere microbiomes for both milkweed species. **(B)** Bar graph showing percentage of bacterial ASVs found in the monarch microbiome that were either unique (only found in caterpillars) or shared with the milkweed phyllosphere and rhizosphere microbiomes. “Generalist” ASVs were found in all three microbiomes.

We compared the overall composition of bacterial communities (i.e., presence/absence of each ASV) across milkweed and monarch microbiomes to determine what proportion of bacterial taxa that are unique vs. shared across microbiomes of the rhizosphere, phyllosphere, or monarch (Figure 5). Overall, 94 ASVs were found in all microbiomes, which is 45.85% of the monarch microbiome, 22.81% of the phyllosphere, and 4.39% of the rhizosphere. Rhizosphere communities have the fewest microbial taxa in common with the other two microbiomes (monarch: 8.51%; phyllosphere: 18.43%), while the monarch (88.78%) and phyllosphere (95.63%) microbiomes both share the majority of their ASVs with the rhizosphere. Interestingly, the monarch microbiome (52.68%) and the phyllosphere (26.21%) share a lower proportion of ASVs. Of the 205 total bacterial ASVs detected in the monarch microbiome, a strikingly low percentage are unique to monarchs or shared with leaves, while in contrast the majority of monarch ASVs were detected in the milkweed rhizosphere (Figure 5). Among the seven bacterial families present in the monarch microbiome, six were also found in the milkweed phyllosphere and rhizosphere and only one was completely unique to monarch communities (Flavobacteriaceae).

## Discussion

Currently, little is known about the microbiomes of milkweeds and associated insect herbivores, particularly how differences in defensive chemical profiles shape surrounding microbial communities (Agrawal et al., 2012). In the current study, first we found that monarch, phyllosphere, and rhizosphere microbiomes differed across milkweed host plant species in terms of taxonomic composition but not overall species richness. Second, monarch feeding did not significantly alter phyllosphere or rhizosphere microbiomes. And third, monarch microbiomes had more in common with the root rhizosphere compared to the leaf phyllosphere. Overall, these results suggest defensive chemical profiles can select for divergent plant and herbivore microbial communities, but we did not find support for our initial prediction that the milkweed species with higher concentrations of cardenolides (i.e., *A. curassavica*) would select for overall lower microbial diversity in the both plant and herbivore associated microbiomes.

Plant species is a distinctive factor known to influence rhizosphere and phyllosphere communities (Costa et al., 2006; Xiong et al., 2021). Although phylogenetically similar hosts tend to harbor similar core sets of microbes (Lajoie and Kembel, 2021) there are distinctive differences across plant species (Pendergast et al., 2013; Laforest-Lapointe et al., 2016; Runge et al., 2021). For example, a recent study found that two closely related tree species (*Acacia heterophylla A. koa*), which speciated 1.4 million years ago have distinct microbiomes with a similar “core” of associated rhizosphere taxa (Le Roux et al., 2021). Not only do plant microbiomes differ across species, but insect herbivore microbiomes also show unique patterns associated with differences in the host plants they feed on as well. Shifts in microbiome diversity and composition linked to diet breadth (i.e., number of unique host plants) have been observed in six closely related *Cephaloleia* species (Blankenchip et al., 2018) and between 146 different caterpillar species feeding on different host plant trees (Li et al., 2021). Interestingly, when both specialist and generalist beetles fed on invasive host plant species changes in their gut microbiomes suggested they were experiencing dysbiosis (Blankenchip et al., 2018). Similarly, the gut microbiome of the Asian longhorned beetle (*Anoplophora glabripennis*) is altered when feeding on different hosts, which in some cases can impact bacterial taxa known to provide digestion and nutritional functions (Scully et al., 2018).

Plant defensive chemistry is one factor that could explain differences in the microbial communities found in various plant tissues and the insects that feed on them. Secondary plant compounds act not only as defensive tools against insect herbivores but can be highly effective against microorganisms as well. Secondary plant compounds may alter the microbial communities of both plants and herbivores, possibly due to antimicrobial effects that select against sensitive taxa while enriching for those capable of degrading or detoxifying plant defensive chemicals (Walenciak et al., 2002; Gao et al., 2003; Keshavan et al., 2005; Davis and Hofstetter, 2012; Raffa, 2014; Hammer and Bowers, 2015; Mason et al., 2015; Pham et al., 2021; Rat et al., 2021). Although this study does not directly test for which plant microbes are affected by secondary plant compounds, our analysis (see Figure 3) identified differentially abundant bacterial families between milkweed species found in the rhizosphere (Dongiaceae, Moraxellaceae, Schlesneriaceae, Sneathiellaceae) and the phyllosphere (Phormidiaceae, Rhizobiaceae); which are potential candidates to further test for interactions with milkweed secondary compounds. Recent studies, through the use of plant mutants, gene knock outs, and growth assays, have shown that secondary plant compounds can have differential toxicity to individual microbes and change community composition (Pang et al., 2021). Antimicrobial effects could therefore influence microbiome assembly if microbes insensitive to defensive compounds survive or those that have detoxification capabilities survive and outcompete other community members.

In the current study, we found no changes in microbial species evenness or richness (Figure 1) but did find differences in community structure between microbiomes associated with *A. syriaca* and *A. curassavica* (Figure 2). These results suggest that differences in plant defensive chemistry are not imposing strong purifying selection that would reduce overall bacterial diversity according to our initial prediction, but instead cause shifts in the composition of communities. Several factors could be contributing to potential direct/indirect effects on microbes that impact beta diversity (composition/structure) but not alpha diversity (species richness/evenness). Cardenolide diversity and concentration are distinct between the two milkweed species used in this study (Rasmann and Agrawal, 2011) and the expression of cardenolides is also tissue specific (Rasmann and Agrawal, 2011). However, *Asclepias* spp. also produce an array of additional secondary plant compounds which can also be unique to species (Agrawal et al., 2009; Araya et al., 2012; de Leão et al., 2020). Therefore the unique defensive profiles of milkweed species could enrich for taxonomically different groups of microbes that are either insensitive to or capable of metabolizing variable amounts and types of phytotoxins, without affecting overall species richness in communities. Differences in host plant physiology could also contribute to shaping overall microbial community composition, such as variation in plant immune responses needed to regulate interactions with microbes, release of organic carbon via rhizodeposits in the roots, and root architecture (Hacquard et al., 2017; Hassan et al., 2019; Pervaiz et al., 2020). For insect herbivores, variation in nutritional content across host plant species (Killiny, 2016; Gallinger and Gross, 2018) is likely to influence insect gut microbial community composition and structure (Hammer and Bowers, 2015). For example, monarchs may be able to digest one plant species more efficiently than another, which could alter availability of carbon or other nutrients utilized by gut microbes. Additionally, our results show feeding on *A. curassavica* reduced monarch growth by 89% compared to *A. syraica* (Supplementary Figure 1), which could also contribute to differences in insect bacterial communities observed across milkweed species via general physiological disruptions or elevated immune responses.

Interestingly, our results show monarch feeding does not impose large top-down effects on microbial communities associated with host plant roots or leaves (Figure 4). In contrast, previous studies show aboveground herbivore feeding by both sap-sucking and chewing herbivores can induce changes in plant associated microbial communities (Humphrey and Whiteman, 2020; French et al., 2021; Malacrinò et al., 2021). For example, aphids can prime systemic plant defense responses, leading to increases in beneficial microbes and reductions in pathogens in the rhizosphere (Lee et al., 2012). Induction of plant defenses in response to herbivore feeding is hypothesized to play a role in changes observed in root and leaf microbiomes (Doornbos et al., 2012). In milkweed, not only are cardenolides induced by herbivore feeding in leaf and root tissues, but microbes have also been shown to induce cardenolide production (Agrawal et al., 2012). However, in our study monarch feeding did not cause restructuring of milkweed microbiomes as we initially predicted. One possible explanation is that a threshold level of insect feeding pressure or stress is needed to cause changes in host plant microbial communities. Here we infested plants with a single 2nd instar monarch larva for 4 days, however, longer feeding duration, multiple individuals and/or larger monarch larva may be needed to induce changes in milkweed bacterial communities. We also did not inoculate milkweed plants with natural soil microbial communities, which may be more responsive to changes in plant physiology induced by herbivore feeding.

Although we did not see top-down affects from monarch feeding, we found a large percent of bacterial taxa were shared between monarchs, the phyllosphere, and rhizosphere. Surprisingly, monarch and rhizosphere microbiomes shared more taxa in common than with the phyllosphere; this was unexpected as monarchs consume large amounts of leaf tissue and constantly walk across leaf surfaces. Interestingly, this could be a broader trend, as two recent studies found similar results. Hannula et al. (2019) found the microbiome of the cabbage moth (*Manestra brassicae*) had greater diversity and resembled the soil microbiomes of intact common dandelion (*Taraxacum officinale)*, while those feeding on detached leaves from the same plants had much lower diversity and resembled the phyllosphere microbiome. Another study, which sampled *Tyria jacobaeae* caterpillars feeding on a species of Asteraceae (*Jacobaea vulgaris*), found that ∼ 25% of the caterpillar’s microbiome was shared with the soil and this trend was consistent across three different habitats (Gomes et al., 2020). One explanation for this trend is that insect frass falls on the soil surface—directly transferring insect microbes and thus making the rhizosphere more like the caterpillar gut microbiome. Alternatively, environmental disturbances including soil splashing up when wet (hitting caterpillars) or caterpillars walking across the soil could provide opportunities for microbial transfer between insect and soil microbiomes (Hannula et al., 2019). Rhizosphere microbiomes are also well known for their expansive mutualistic functions (Khan et al., 2021), which might provide a reservoir of potential microbial partners that are more quickly assimilated into caterpillar microbiomes. However, it is generally unclear what mechanisms or routes of microbial transfer contribute to the high proportion of shared microbes between leaf chewing herbivores and host plant associated rhizosphere or soil microbiomes. Further research is needed to identify how chewing herbivores acquire microbes from their environment and if there are differences depending on type of microbe examined within the broader community (e.g., bacteria vs. fungi). As more studies characterize the interconnected microbial communities of plants and insect herbivores it may be possible to develop advanced traceability analysis (e.g., Knights et al., 2011) that will allow researchers to determine from what environmental sources shared microbes originate and how microbes are moving between communities associated with roots, leaves and insect tissues.

One potential outcome of environmental microbial crossover or horizonal transfer between plants and insects may be unique ecological interactions. In this study, the most dominant bacterial family associated with milkweed leaves was Anaplasmataceae (Table 1 and Figure 3), specifically the genus *Wolbachia*. *Wolbachia* is an intracellular symbiont, best known for its ubiquitous presence across terrestrial arthropods where it commonly acts as a reproductive manipulator (Kaur et al., 2021). However, *Wolbachia* can also form mutualistic relationships with the host. Previous research has shown *Wolbachia* nutritionally supplementing B group vitamins to it host (Ju et al., 2020) and mediating essential functions for leaf mining moths to impose a “green-island” phenotype that keeps leaf tissue photosynthetically active as it is being fed on (Gutzwiller et al., 2015; Zhang et al., 2018). While it may seem unusual to find an intracellular insect symbiont associated with milkweed leaf tissue, plant mediated horizontal transfer of *Wolbachia* has been shown in *Crioceris* leaf beetles (Kolasa et al., 2017) and whiteflies (Li et al., 2017). Endophytic Anaplasmataceae microbes are also found in *Miscanthus sinensis* plants (Cope-Selby et al., 2017). The presence of *Wolbachia* in milkweed is interesting and should be further explored, particularly whether *Wolbachia* are horizontally transferred and what role they may play in monarch and milkweed biology (i.e., pathogens/mutualists).

## Conclusion

Research on monarch and milkweed microbiomes has been limited; our study is one of the first to characterize the microbial communities of both. We found that the composition of the monarch microbiome, phyllosphere, and rhizosphere are influenced by milkweed plant species and that 88.78% of monarch bacterial taxa are shared with the rhizosphere. Future studies are needed that focus on the functional characterization of the microbiome and test microbial metabolic capability to break down key milkweed chemical defenses (e.g., cardiac glycosides). Specifically, bioassays that screen for detoxification functions in microbes associated with milkweeds and monarchs will help determine the extent to which plant defensive chemicals impose selection on communities and potentially select for taxa that aide in monarch digestion of toxic plant material. Both metagenomics and metatranscriptomics are needed to move beyond characterizing diversity to identifying expressed genes and biological pathways of interest. Combined metagenomics and metatranscriptomics approaches will also provide a route to assess microbe-microbe interactions, which is not addressed in most contemporary studies.

## Data Availability Statement

The data is publicly available here: https://www.ncbi.nlm.nih.gov/bioproject/PRJNA786874, accession PRJNA786874.

## Author Contributions

TH and LE conceived and designed the study, wrote, and revised all drafts of the manuscript. TH collected all data, performed all statistical analyses, and developed all figures. Both authors contributed to manuscript revisions, read, and approved the final manuscript.

## Conflict of Interest

The authors declare that the research was conducted in the absence of any commercial or financial relationships that could be construed as a potential conflict of interest.

## Publisher’s Note

All claims expressed in this article are solely those of the authors and do not necessarily represent those of their affiliated organizations, or those of the publisher, the editors and the reviewers. Any product that may be evaluated in this article, or claim that may be made by its manufacturer, is not guaranteed or endorsed by the publisher.

## Footnotes

1
https://github.itap.purdue.edu/LaramyEndersGroup/Milkweed-Monarch-Microbiomes
