# Supplementary material for: Host Plant Species Influences the Composition of Milkweed and Monarch Microbiomes
Source: Front Microbiol. 2022 Feb 24;13:840078. doi: 10.3389/fmicb.2022.840078 (PMC8908431; doi:10.3389/fmicb.2022.840078)
Supplement: Supplementary file 1 [file Data_Sheet_1.docx]

**Supplemental Tables**

**Supplemental Table 1:** Sequencing summary.

|  | Total paired reads | # of ASVs |
| --- | --- | --- |
| Initial reads | 4,371,882 | NA |
| After adapter/primer clipping and low quality read removal | 3,300,444 | NA |
| Dada2 quality filtering | 3,246,295 | NA |
| Error correction, merged F and R reads, length filtering | 3,086,562 | NA |
| Chimera removal | 2,816,610 | 9,489 |
| Non-target sequence removal | 1,524,064 | 9,319 |
| Likely contaminant removal | 1,515,690 | 9,313 |
| Ambiguous feature removal | 1,317,237 | 6,915 |
| Kept taxa found more than 3 times in at least 5% of the samples | 1,211,088 | 2,164 |

**Supplemental Table 2:** Summary of results from Kruskal-Wallis tests on alpha diversity measures (observed species richness, evenness, Shannon, Simpson) compared across plant species and insect presence in the different microbiome types (insect/monarch, milkweed phyllosphere & rhizosphere).

**Observed Species Richness**

|  | Degrees of freedom | Sample size | H statistic | Effect size | Magnitude | Pr(>F) |
| --- | --- | --- | --- | --- | --- | --- |
| **Insect** |  |  |  |  |  |  |
| Plant Species | 1 | 13 | 0.005116096 | -0.09044399 | Moderate | 0.943 |
|  |  |  |  |  |  |  |
| **Phyllosphere** |  |  |  |  |  |  |
| Plant Species | 1 | 28 | 0.4750176 | -0.02019163 | Small | 0.491 |
| Insect Presence | 1 | 28 | 0.2111189 | -0.03034158 | Small | 0.646 |
|  |  |  |  |  |  |  |
| **Rhizosphere** |  |  |  |  |  |  |
| Plant Species | 1 | 28 | 1.319493 | 0.0122882 | Small | 0.251 |
| Insect Presence | 1 | 28 | 0.356791 | -0.02473881 | Small | 0.55 |

**Evenness**

|  | Degrees of freedom | Sample size | H statistic | Effect size | Magnitude | Pr(>F) |
| --- | --- | --- | --- | --- | --- | --- |
| **Insect** |  |  |  |  |  |  |
| Plant Species | 1 | 13 | 1 | 0 | Small | 0.317 |
|  |  |  |  |  |  |  |
| **Phyllosphere** |  |  |  |  |  |  |
| Plant Species | 1 | 28 | 0.6840253 | -0.01215287 | Small | 0.408 |
| Insect Presence | 1 | 28 | 4.087262 | 0.1187409 | Moderate | 0.0432 |
|  |  |  |  |  |  |  |
| **Rhizosphere** |  |  |  |  |  |  |
| Plant Species | 1 | 28 | 2.028853 | 0.03957127 | Small | 0.154 |
| Insect Presence | 1 | 28 | 0.6840253 | -0.01215287 | Small | 0.408 |

**Shannon Diversity Index (H)**

|  | Degrees of freedom | Sample size | H statistic | Effect size | Magnitude | Pr(>F) |
| --- | --- | --- | --- | --- | --- | --- |
| **Insect** |  |  |  |  |  |  |
| Plant Species | 1 | 13 | 0.3265306 | -0.06122449 | Moderate | 0.568 |
|  |  |  |  |  |  |  |
| **Phyllosphere** |  |  |  |  |  |  |
| Plant Species | 1 | 28 | 0.07600281 | -0.03553835 | Small | 0.783 |
| Insect Presence | 1 | 28 | 2.890218 | 0.0727007 | Moderate | 0.0891 |
|  |  |  |  |  |  |  |
| **Rhizosphere** |  |  |  |  |  |  |
| Plant Species | 1 | 28 | 2.440535 | 0.05540519 | Small | 0.118 |
| Insect Presence | 1 | 28 | 0.5404645 | -0.01767444 | Small | 0.462 |

**Simpson Diversity Index**

|  | Degrees of freedom | Sample size | H statistic | Effect size | Magnitude | Pr(>F) |
| --- | --- | --- | --- | --- | --- | --- |
| **Insect** |  |  |  |  |  |  |
| Plant Species | 1 | 13 | 1 | 0 | Small | 0.317 |
|  |  |  |  |  |  |  |
| **Phyllosphere** |  |  |  |  |  |  |
| Plant Species | 1 | 28 | 0.6840253 | -0.01215287 | Small | 0.408 |
| Insect Presence | 1 | 28 | 4.087262 | 0.1187409 | Moderate | 0.0432 |
|  |  |  |  |  |  |  |
| **Rhizosphere** |  |  |  |  |  |  |
| Plant Species | 1 | 28 | 2.161858 | 0.04468684 | Small | 0.141 |
| Insect Presence | 1 | 28 | 0.7621393 | -0.009148487 | Small | 0.383 |

**Supplemental Table 3:** Summary of PERMANOVA of beta diversity metrics (Bray-Curtis dissimilarity, Jaccard, unweighted unifrac, weighted unifrac) comparing plant species for each microbiome type (insect/monarch, milkweed phyllosphere & rhizosphere) and +/- insect presence for milkweed microbiomes.

**Bray Curtis Dissimilarity**

|  | Degrees of freedom | Sum of Squares | Means Squared | F statistic | R^2^ | Pr(>F) |
| --- | --- | --- | --- | --- | --- | --- |
|  |  |  |  |  |  |  |
| **Insect** |  |  |  |  |  |  |
| Plant Species | 1 | 0.5415 | 0.54153 | 2.2529 | 0.16999 | 0.002 |
| Residuals | 11 | 2.6441 | 0.24038 |  | 0.83001 |  |
|  |  |  |  |  |  |  |
| **Phyllosphere** |  |  |  |  |  |  |
| Plant Species | 1 | 0.8497 | 0.84970 | 2.99717 | 0.10346 | 0.001 |
| Insect Presence | 1 | 0.2960 | 0.29600 | 1.04409 | 0.03604 | 0.390 |
| Plant Species x Insect Presence | 1 | 0.2633 | 0.26328 | 0.92868 | 0.03206 | 0.600 |
| Residuals | 24 | 6.8040 |  |  | 0.82844 |  |
|  |  |  |  |  |  |  |
| **Rhizosphere** |  |  |  |  |  |  |
| Plant Species | 1 | 0.5476 | 0.54756 | 4.1747 | 0.13938 | 0.001 |
| Insect Presence | 1 | 0.1029 | 0.10285 | 0.7841 | 0.02618 | 0.747 |
| Plant Species x Insect Presence | 1 | 0.1303 | 0.13034 | 0.9937 | 0.03318 | 0.408 |
| Residuals | 24 | 3.1479 | 0.13116 |  | 0.80127 |  |

**Jaccard**

|  | Degrees of freedom | Sum of Squares | Means Squared | F statistic | R^2^ | Pr(>F) |
| --- | --- | --- | --- | --- | --- | --- |
|  |  |  |  |  |  |  |
| **Insect** |  |  |  |  |  |  |
| Plant Species | 1 | 0.5698 | 0.56981 | 1.7254 | 0.13559 | 0.001 |
| Residuals | 11 | 3.6328 | 0.33025 |  | 0.86441 |  |
|  |  |  |  |  |  |  |
| **Phyllosphere** |  |  |  |  |  |  |
| Plant Species | 1 | 0.7814 | 0.78145 | 2.13372 | 0.07604 | 0.001 |
| Insect Presence | 1 | 0.3625 | 0.36246 | 0.98968 | 0.03527 | 0.507 |
| Plant Species x Insect Presence | 1 | 0.3427 | 0.34269 | 0.93569 | 0.03335 | 0.679 |
| Residuals | 24 | 8.7897 | 0.36624 |  | 0.85534 |  |
|  |  |  |  |  |  |  |
| **Rhizosphere** |  |  |  |  |  |  |
| Plant Species | 1 | 0.7022 | 0.70221 | 3.10564 | 0.10729 | 0.001 |
| Insect Presence | 1 | 0.1958 | 0.19578 | 0.86587 | 0.02991 | 0.707 |
| Plant Species x Insect Presence | 1 | 0.2204 | 0.22041 | 0.97478 | 0.03368 | 0.435 |
| Residuals | 24 | 5.4266 | 0.22611 |  | 0.82912 |  |

**Unweighted Unifrac**

|  | Degrees of freedom | Sum of Squares | Means Squared | F statistic | R^2^ | Pr(>F) |
| --- | --- | --- | --- | --- | --- | --- |
|  |  |  |  |  |  |  |
| **Insect** |  |  |  |  |  |  |
| Plant Species | 1 | 0.39958 | 0.39958 | 2.0289 | 0.15572 | 0.03 |
| Residuals | 11 | 2.16638 | 0.19694 |  | 0.84428 |  |
|  |  |  |  |  |  |  |
| **Phyllosphere** |  |  |  |  |  |  |
| Plant Species | 1 | 0.2865 | 0.28650 | 1.27388 | 0.04678 | 0.139 |
| Insect Presence | 1 | 0.2443 | 0.24433 | 1.08637 | 0.03990 | 0.295 |
| Plant Species x Insect Presence | 1 | 0.1954 | 0.19535 | 0.86861 | 0.03190 | 0.660 |
| Residuals | 24 | 5.3976 | 0.22490 |  | 0.88142 |  |
|  |  |  |  |  |  |  |
| **Rhizosphere** |  |  |  |  |  |  |
| Plant Species | 1 | 0.28369 | 0.283693 | 3.2158 | 0.11084 | 0.001 |
| Insect Presence | 1 | 0.07985 | 0.079851 | 0.9052 | 0.03120 | 0.595 |
| Plant Species x Insect Presence | 1 | 0.07874 | 0.078737 | 0.8925 | 0.03076 | 0.604 |
| Residuals | 24 | 2.11723 | 0.088218 |  | 0.82720 |  |

**Weighted Unifrac**

|  | Degrees of freedom | Sum of Squares | Means Squared | F statistic | R^2^ | Pr(>F) |
| --- | --- | --- | --- | --- | --- | --- |
|  |  |  |  |  |  |  |
| **Insect** |  |  |  |  |  |  |
| Plant Species | 1 | 0.09606 | 0.096064 | 1.8393 | 0.14326 | 0.062 |
| Residuals | 11 | 0.57450 | 0.052227 |  | 0.85674 |  |
|  |  |  |  |  |  |  |
| **Phyllosphere** |  |  |  |  |  |  |
| Plant Species | 1 | 0.11362 | 0.113617 | 2.3567 | 0.08243 | 0.006 |
| Insect Presence | 1 | 0.06479 | 0.064795 | 1.3440 | 0.04701 | 0.165 |
| Plant Species x Insect Presence | 1 | 0.04285 | 0.042855 | 0.8889 | 0.03109 | 0.538 |
| Residuals | 24 | 1.15706 | 0.048211 |  | 0.83947 |  |
|  |  |  |  |  |  |  |
| **Rhizosphere** |  |  |  |  |  |  |
| Plant Species | 1 | 0.028848 | 0.0288483 | 4.2559 | 0.14294 | 0.001 |
| Insect Presence | 1 | 0.004962 | 0.0049620 | 0.7320 | 0.02459 | 0.801 |
| Plant Species x Insect Presence | 1 | 0.005324 | 0.0053236 | 0.7854 | 0.02638 | 0.737 |
| Residuals | 24 | 0.162681 | 0.0067784 |  | 0.80609 |  |

**Supplemental Table 4:** Summary of PERMDISP tests of beta diversity metrics (Bray-Curtis dissimilarity, Jaccard, unweighted unifrac, weighted unifrac) comparing plant species for each microbiome type (insect/monarch, milkweed phyllosphere & rhizosphere) and +/- insect presence for milkweed microbiomes.

**Bray-Curtis Dissimilarity**

|  | Degrees of freedom | Sum of Squares | Means Squared | F statistic | Number of permutations | Pr(>F) |
| --- | --- | --- | --- | --- | --- | --- |
|  |  |  |  |  |  |  |
| **Insect** |  |  |  |  |  |  |
| Plant Species | 1 | 0.003204 | 0.0032037 | 0.4109 | 999 | 0.505 |
| Residuals | 11 | 0.085765 | 0.0077968 |  |  |  |
|  |  |  |  |  |  |  |
| **Phyllosphere** |  |  |  |  |  |  |
| Plant Species | 1 | 0.00123 | 0.0012304 | 0.2435 | 999 | 0.609 |
| Residuals | 26 | 0.13137 | 0.0050526 |  |  |  |
| Insect Presence | 1 | 0.000261 | 0.0002613 | 0.0803 | 999 | 0.775 |
| Residuals | 26 | 0.084561 | 0.0032523 |  |  |  |
|  |  |  |  |  |  |  |
| **Rhizosphere** |  |  |  |  |  |  |
| Plant Species | 1 | 0.000886 | 0.0008860 | 0.4151 | 999 | 0.592 |
| Residuals | 26 | 0.055496 | 0.0021345 |  |  |  |
| Insect Presence | 1 | 0.004710 | 0.0047103 | 2.1118 | 999 | 0.165 |
| Residuals | 26 | 0.057992 | 0.0022305 |  |  |  |

**Jaccard**

|  | Degrees of freedom | Sum of Squares | Means Squared | F statistic | Number of permutations | Pr(>F) |
| --- | --- | --- | --- | --- | --- | --- |
|  |  |  |  |  |  |  |
| **Insect** |  |  |  |  |  |  |
| Plant Species | 1 | 0.001645 | 0.0016453 | 0.4237 | 999 | 0.529 |
| Residuals | 11 | 0.042711 | 0.0038828 |  |  |  |
|  |  |  |  |  |  |  |
| **Phyllosphere** |  |  |  |  |  |  |
| Plant Species | 1 | 0.000423 | 0.00042308 | 0.1991 | 999 | 0.637 |
| Residuals | 26 | 0.055256 | 0.00212523 |  |  |  |
| Insect Presence | 1 | 0.000100 | 0.00010005 | 0.0777 | 999 | 0.81 |
| Residuals | 26 | 0.033497 | 0.00128835 |  |  |  |
|  |  |  |  |  |  |  |
| **Rhizosphere** |  |  |  |  |  |  |
| Plant Species | 1 | 0.000432 | 0.00043177 | 0.2836 | 999 | 0.65 |
| Residuals | 26 | 0.039590 | 0.00152268 |  |  |  |
| Insect Presence | 1 | 0.003416 | 0.0034163 | 2.4031 | 999 | 0.128 |
| Residuals | 26 | 0.036962 | 0.0014216 |  |  |  |

**Unweighted Unifrac**

|  | Degrees of freedom | Sum of Squares | Means Squared | F statistic | Number of permutations | Pr(>F) |
| --- | --- | --- | --- | --- | --- | --- |
|  |  |  |  |  |  |  |
| **Insect** |  |  |  |  |  |  |
| Plant Species | 1 | 0.004400 | 0.0043996 | 0.7373 | 999 | 0.359 |
| Residuals | 11 | 0.065639 | 0.0059672 |  |  |  |
|  |  |  |  |  |  |  |
| **Phyllosphere** |  |  |  |  |  |  |
| Plant Species | 1 | 0.000135 | 0.0001348 | 0.023 | 999 | 0.886 |
| Residuals | 26 |  |  |  |  |  |
| Insect Presence | 1 | 0.005938 | 0.0059381 | 1.396 | 999 | 0.286 |
| Residuals | 26 | 0.110591 | 0.0042535 |  |  |  |
|  |  |  |  |  |  |  |
| **Rhizosphere** |  |  |  |  |  |  |
| Plant Species | 1 | 0.000137 | 0.00013704 | 0.0594 | 999 | 0.856 |
| Residuals | 26 | 0.059988 | 0.00230725 |  |  |  |
| Insect Presence | 1 | 0.004011 | 0.0040105 | 2.0774 | 999 | 0.167 |
| Residuals | 26 | 0.050196 | 0.0019306 |  |  |  |

**Weighted Unifrac**

|  | Degrees of freedom | Sum of Squares | Means Squared | F statistic | Number of permutations | Pr(>F) |
| --- | --- | --- | --- | --- | --- | --- |
|  |  |  |  |  |  |  |
| **Insect** |  |  |  |  |  |  |
| Plant Species | 1 | 0.0028269 | 0.0028269 | 0.9912 | 999 | 0.318 |
| Residuals | 11 | 0.0313705 | 0.0028519 |  |  |  |
|  |  |  |  |  |  |  |
| **Phyllosphere** |  |  |  |  |  |  |
| Plant Species | 1 | 0.000018 | 0.00001777 | 0.0077 | 999 | 0.931 |
| Residuals | 26 | 0.060036 | 0.00230907 |  |  |  |
| Insect Presence | 1 | 0.000925 | 0.00092478 | 0.4706 | 999 | 0.492 |
| Residuals | 26 | 0.051096 | 0.00196525 |  |  |  |
|  |  |  |  |  |  |  |
| **Rhizosphere** |  |  |  |  |  |  |
| Plant Species | 1 | 0.0000650 | 0.0000650 | 0.1769 | 999 | 0.745 |
| Residuals | 26 | 0.0095549 | 0.0003675 |  |  |  |
| Insect Presence | 1 | 0.0004736 | 0.00047356 | 1.1009 | 999 | 0.355 |
| Residuals | 26 | 0.0111836 | 0.00043014 |  |  |  |

**Supplemental Table 5:** Significant differences in the phyllosphere and rhizosphere microbiome composition between host plant species as determined using ANCOM. The listed cutoff range (0.6 -0.9) indicates a more relaxed to conservative false discovery rate.

|  | Family | Plant species | W | Centered log-ratio mean differences | Detected at 90% cutoff | Detected at 80% cutoff | Detected at 70% cutoff | Detected at 60% cutoff |
| --- | --- | --- | --- | --- | --- | --- | --- | --- |
| Phyllosphere |  |  |  |  |  |  |  |  |
|  | Rhizobiaceae | A. curassavica | 53 | -1.0785 | No | Yes | Yes | Yes |
|  | Phormidiaceae | A. syriaca | 47 | 0.1246 | No | No | Yes | Yes |
| Rhizosphere |  |  |  |  |  |  |  |  |
|  | Moraxellaceae | A. curassavica | 219 | -0.4739 | Yes | Yes | Yes | Yes |
|  | Dongiaceae | A. curassavica | 175 | -1.5347 | No | No | Yes | Yes |
|  | Schlesneriaceae | A. curassavica | 155 | -1.1993 | No | No | No | Yes |
|  | Sneathiellaceae | A. syriaca | 150 | 0.6306 | No | No | No | Yes |

**Supplemental Table 6:** Results of Welch two sample t-test comparing the change in monarch larval weights between caterpillars that fed on *A. curassavica* and *A. syriaca*

| T-value | Degrees of freedom | 95% confidence interval | *A. curassavica* mean | *A. syriaca* mean | Pr(T < t) |
| --- | --- | --- | --- | --- | --- |
| -4.0744 | 8.8961 | [Infinity, 0.007384127] | 0.008233333 | 0.021671429 | 0.001425 |

**Supplemental Figures**

**
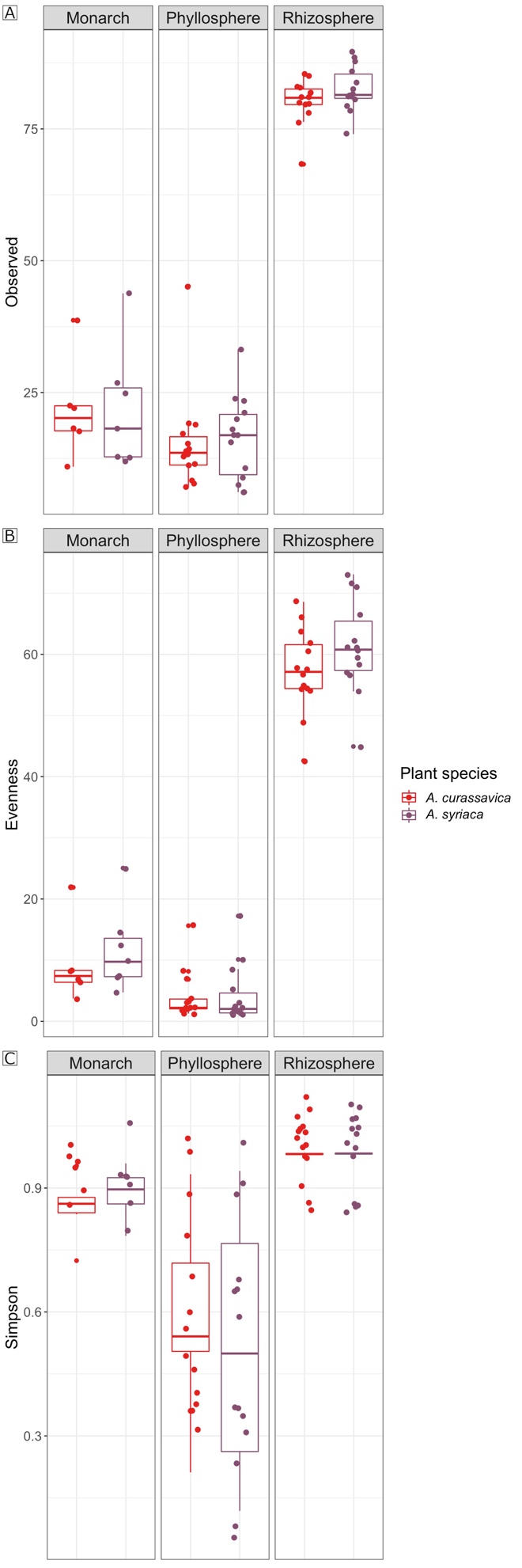
**

**Supplemental Figure 1:** Comparison of alpha diversity metrics of microbial communities associated with two milkweed species (roots/rhizosphere soil, leaves/phyllosphere) and monarch larva after feeding on the different milkweed species. (A) observed species richness, (B) species evenness, and (C) Simpson diversity.


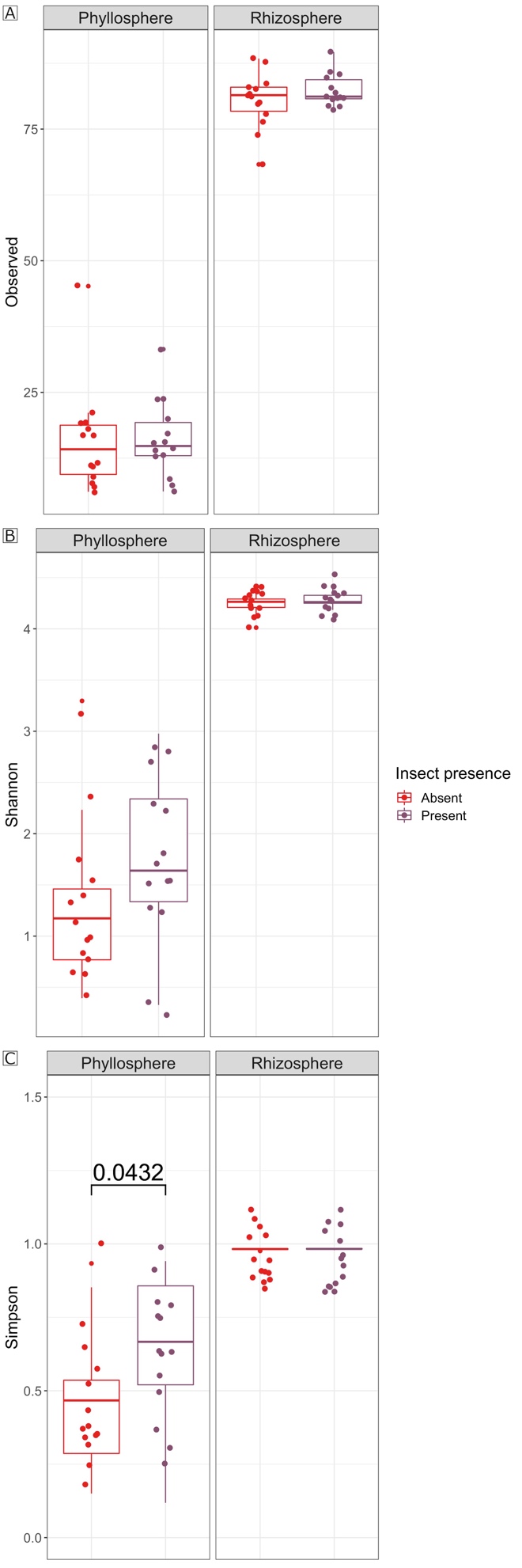


**Supplemental Figure 2:** Comparison of alpha diversity metrics of microbial communities associated with two milkweed species (roots/rhizosphere soil, leaves/phyllosphere) from plants infested with monarch larva and un-infested controls. (A) observed species richness, (B) Shannon diversity, and (C) Simpson diversity.

**
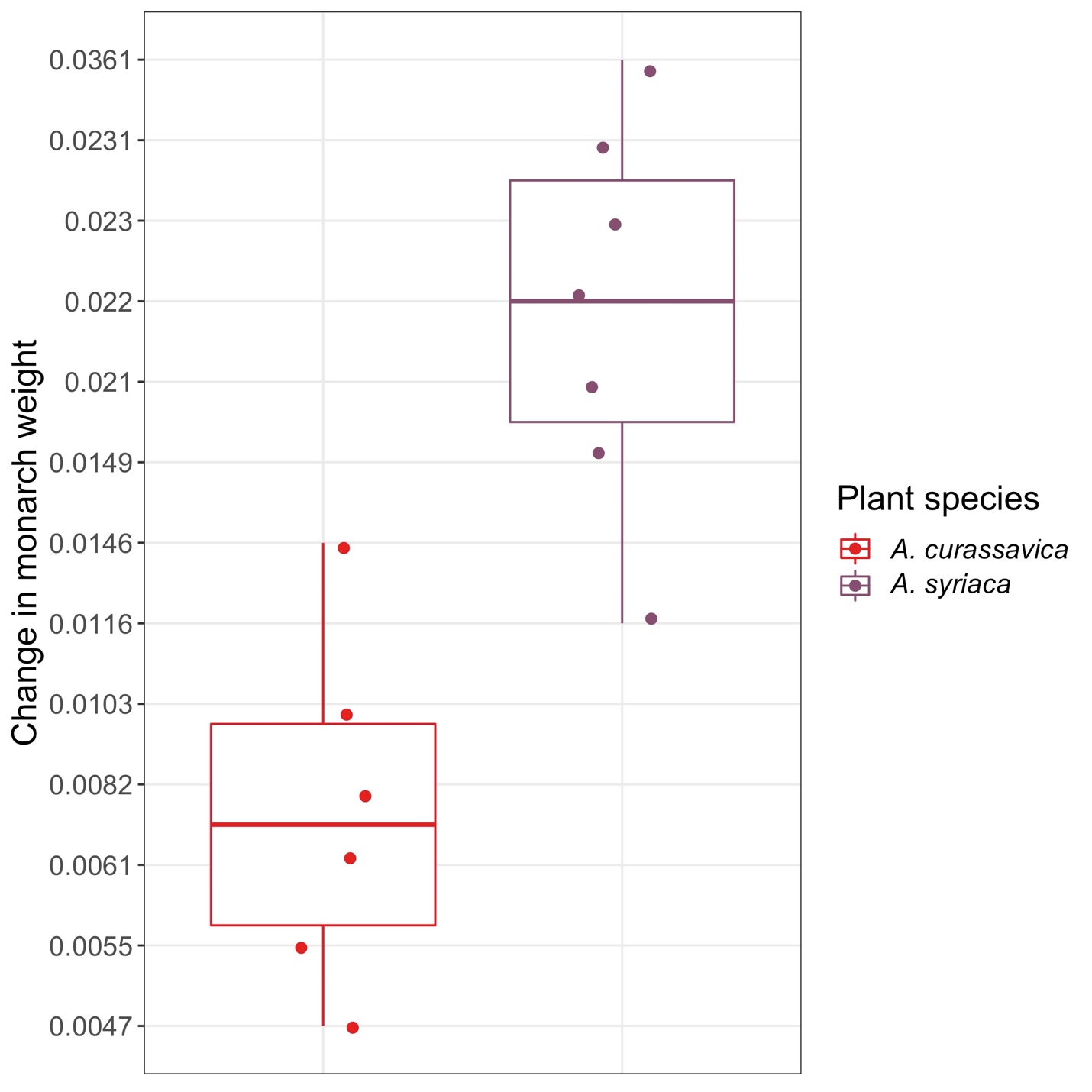
**

**Supplemental Figure 3:** The change in monarch larval weights (final weight – initial weight) between caterpillars that fed on *A. curassavica* and *A. syriaca* (p-value = 0.001425)*.*
